# Growth of single-crystalline cobalt silicide nanowires and their field emission property

**DOI:** 10.1186/1556-276X-8-308

**Published:** 2013-07-03

**Authors:** Chi-Ming Lu, Han-Fu Hsu, Kuo-Chang Lu

**Affiliations:** 1 Department of Materials Science and Engineering, National Cheng Kung University, Tainan 701, Taiwan; 2 Center for Micro/Nano Science and Technology, National Cheng Kung University, Tainan 701, Taiwan

**Keywords:** CVD, Cobalt silicide, Nanowires, Single crystalline, Field emission

## Abstract

In this work, cobalt silicide nanowires were synthesized by chemical vapor deposition processes on Si (100) substrates with anhydrous cobalt chloride (CoCl_2_) as precursors. Processing parameters, including the temperature of Si (100) substrates, the gas flow rate, and the pressure of reactions were varied and studied; additionally, the physical properties of the cobalt silicide nanowires were measured. It was found that single-crystal CoSi nanowires were grown at 850°C ~ 880°C and at a lower gas flow rate, while single-crystal Co_2_Si nanowires were grown at 880°C ~ 900°C. The crystal structure and growth direction were identified, and the growth mechanism was proposed as well. This study with field emission measurements demonstrates that CoSi nanowires are attractive choices for future applications in field emitters.

## Background

Possessing low resistivity and excellent compatibility with conventional silicon device processing, transition metal silicide nanowires have been widely studied [1-5]. Compared with silicon nanowires (NWs), fabricating free-standing silicide NWs is more complicated since metal silicides have lots of phases. In terms of methods, the synthesis of free-standing silicide NWs can be divided into four classifications, which are silicidation of silicon nanowires [6-11], delivery of silicon to metal films [12-16], reactions between transition metal sources and silicon substrates [17-22], and simultaneous metal and silicon delivery [23-25]. Cobalt silicide nanowires have many relatively good characteristics, including low resistivity, good thermal stability, appropriate work function, and compatibility with current processing of Si devices. There are three main methods for synthesizing CoSi NWs, including reactions of CoCl_2_ with silicon substrates by chemical vapor deposition (CVD) processes [26-28], cobalt silicide nanocables grown on Co films [29], and CVD with single-source precursors [30]. In this work, we synthesized cobalt silicide nanowires through CVD processes and changed and studied the effects of several critical processing parameters. Additionally, we conducted scanning electron microscopy (SEM) and transmission electron microscopy (TEM) analyses for identifying the structure and composition of the resultant products and investigating their growth mechanisms. Also, the electrical properties of the nanosilicides were measured and discussed for potential applications.

## Methods

In our study, we synthesized cobalt silicide nanowires by CVD processes using single-crystal Si (100) wafers of native oxide as substrates, anhydrous cobalt chloride powders (97%) as precursors, and Ar gas (99.99%) with H_2_ gas (15%) as carrier gases. The metal sources were put in the upstream zone where the temperature was 610°C, while the silicon (100) substrates were put in the downstream zone, the temperature range of which was 750°C ~ 900°C. To understand the factors that influence the growth of cobalt silicide nanowires, we conducted experiments with different substrate temperatures, vapor pressures, and gas flow rates. SEM was utilized for the morphology of the nanowires, and TEM analysis was conducted for structure identification and atomic resolution imaging of the nanowires.

## Results and discussion

**Figure 1:**
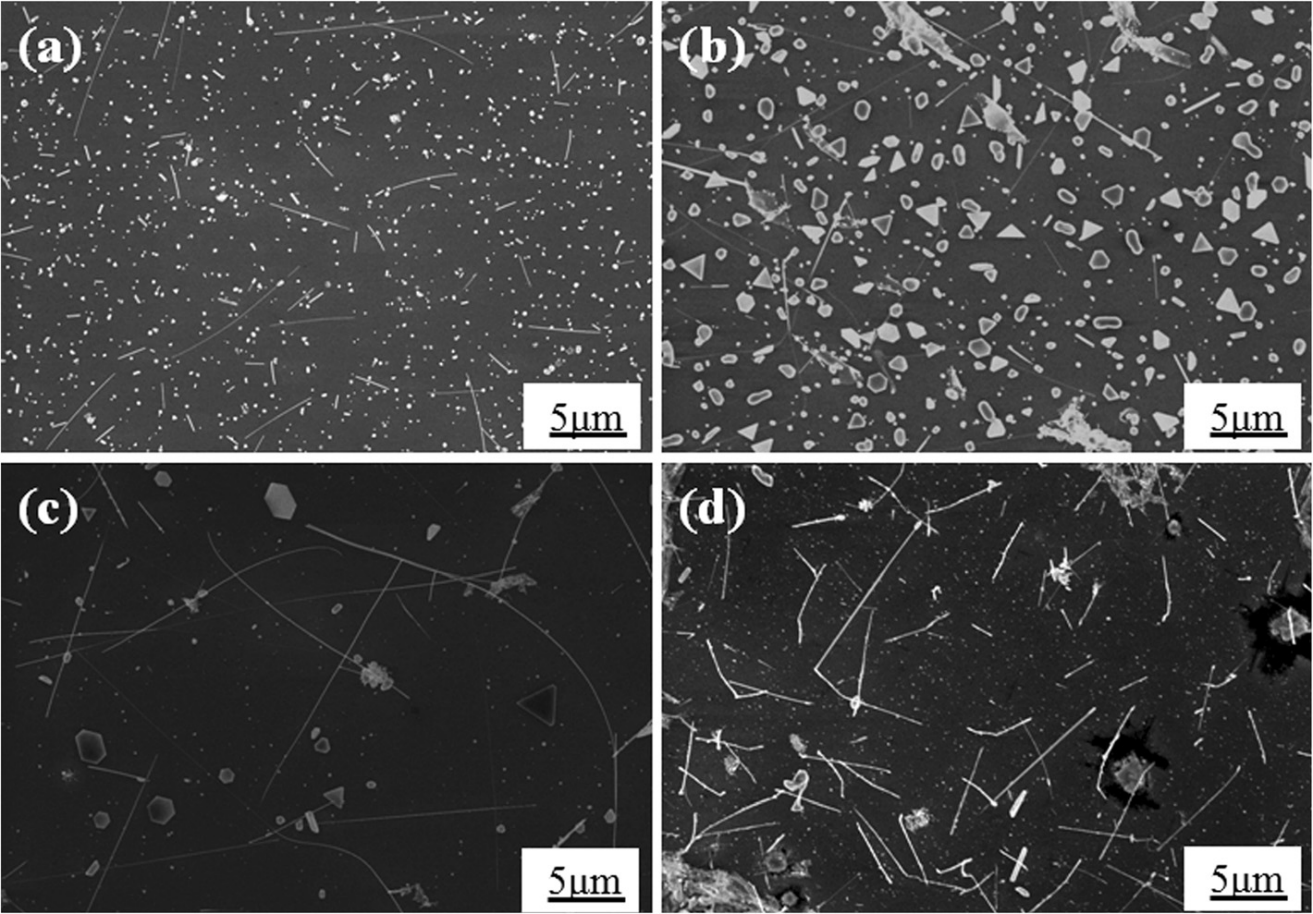
**SEM images of as-synthesized nanowires.** At silicon substrate temperatures of **(a)** 750°C ~ 800°C, **(b)** 800°C ~ 850°C, **(c)** 850°C ~ 880°C, and **(d)** 880°C ~ 900°C, respectively.

**Figure 2:**
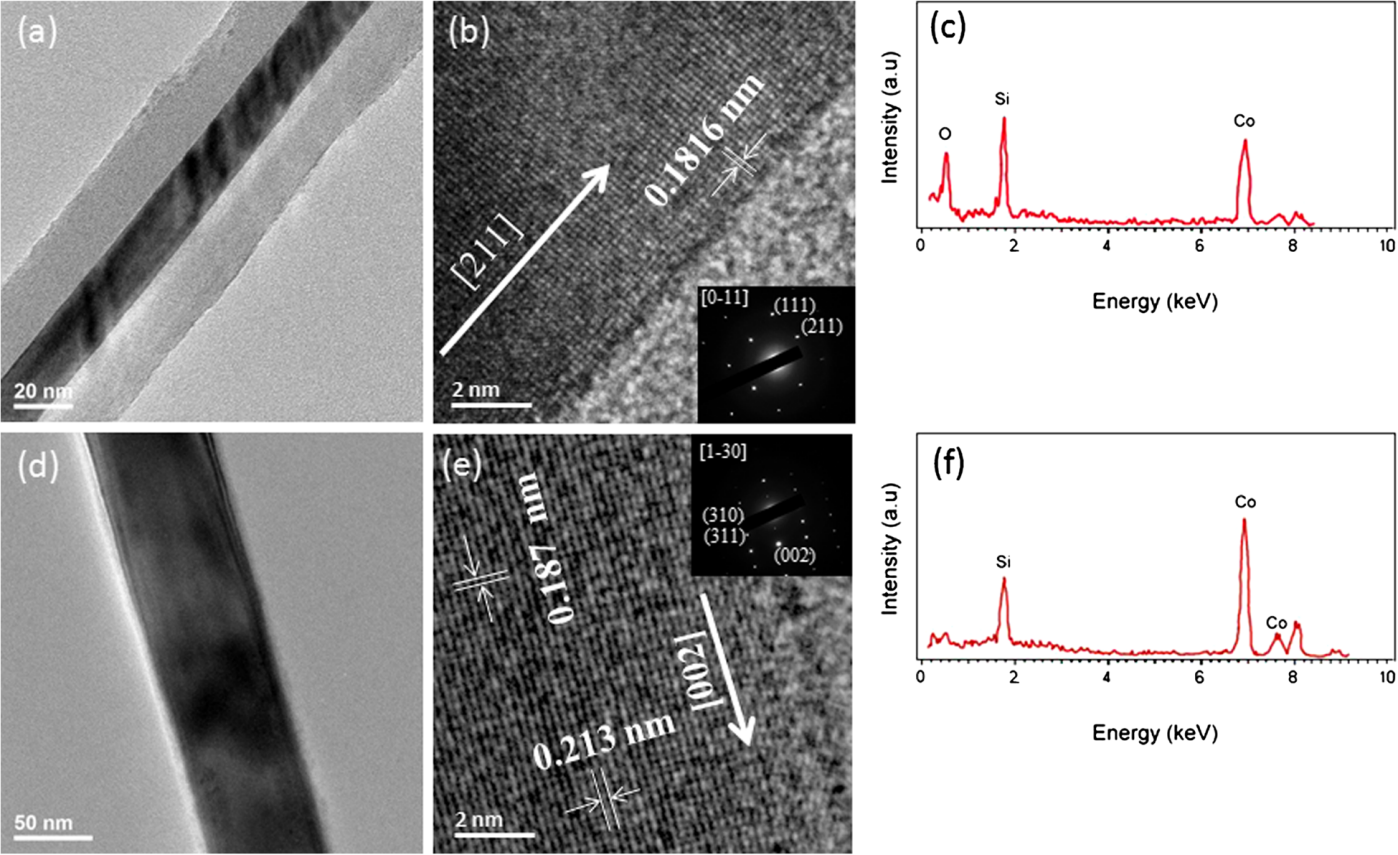
**TEM images and EDS spectra of cobalt silicide nanowires. (a)** Low-magnification, **(b)** high-resolution TEM images and **(c)** EDS spectrum of CoSi nanowires grown at 850°C ~ 880°C. The inset in (b) shows the corresponding selected area diffraction pattern with a zone axis of [0-11]. **(d)** Low-magnification, **(e)** high-resolution TEM images and **(f)** EDS spectrum of Co_2_Si nanowires grown at 880°C ~ 900°C. The inset in **(e)** shows the corresponding selected area diffraction pattern with a zone axis of [1-30].

In our experiments, we varied some processing parameters to investigate how they affect the growth and morphology of the cobalt silicide nanowires. Firstly, we focused on the effect of different substrate temperatures as shown in the SEM images of Figure 1a,b,c,d. Figure 1a shows the case with the substrate temperature of 750°C ~ 800°C, where many nanoparticles and few nanowires were found on silicon substrates. Figure 1b shows the case with the substrate temperature of 800°C ~ 850°C, where there were many nanoparticles larger in size than those found in Figure 1a and few nanowires on silicon substrates. When we increased the substrate temperature to 850°C ~ 880°C as shown in Figure 1c, lots of nanowires of about 15 ~ 20 μm in length and few larger nanoparticles appeared. Figure 1d shows the case with the substrate temperature of 880°C ~ 900°C, where on silicon substrates, we can see many nanowires as well but they are of different morphologies as compared in Figure 1c. For further investigation on the atomic structures of the nanowires, we conducted TEM analysis as shown in Figure 2. It has been confirmed that the nanowires on 850°C ~ 880°C substrates are single-crystal CoSi nanowires with 10 ~ 20 nm SiOx as an outer layer as shown in Figure 2a. The high-resolution TEM image in Figure 2b and the corresponding selected area diffraction pattern in its inset show that the single-crystal CoSi nanowire has a cubic B20-type structure with a lattice constant of 0.4446 nm; also, the growth direction is [211], and the interplanar distance of (211) is 0.1816 nm. Figure 2c is an energy-dispersive X-ray spectroscopy (EDS) spectrum for the nanowires showing that in addition to cobalt and silicon, there is also oxygen and that the atomic percentage ratio for Co/Si/O = 5:8:12. Since the core structure has been identified to be CoSi, all these results reasonably indicate that the shell material is amorphous silicon oxide. On 880°C ~ 900°C substrates, Figure 2d shows a single-crystal Co_2_Si nanowire without surface oxide. The high-resolution TEM image in Figure 2e and the corresponding selected area diffraction pattern in its inset show that the single-crystal Co_2_Si nanowire has an orthorhombic structure with [002] growth direction and lattice constants of *a* = 0.4918 nm, *b* = 0.7109 nm, and *c* = 0.3738 nm and that the interplanar distances of plane (002) and plane (310) are 0.187 and 0.213 nm, respectively. Figure 2f shows an EDS spectrum indicating that the ratio of Co and Si is close to 2:1.

**Figure 3:**
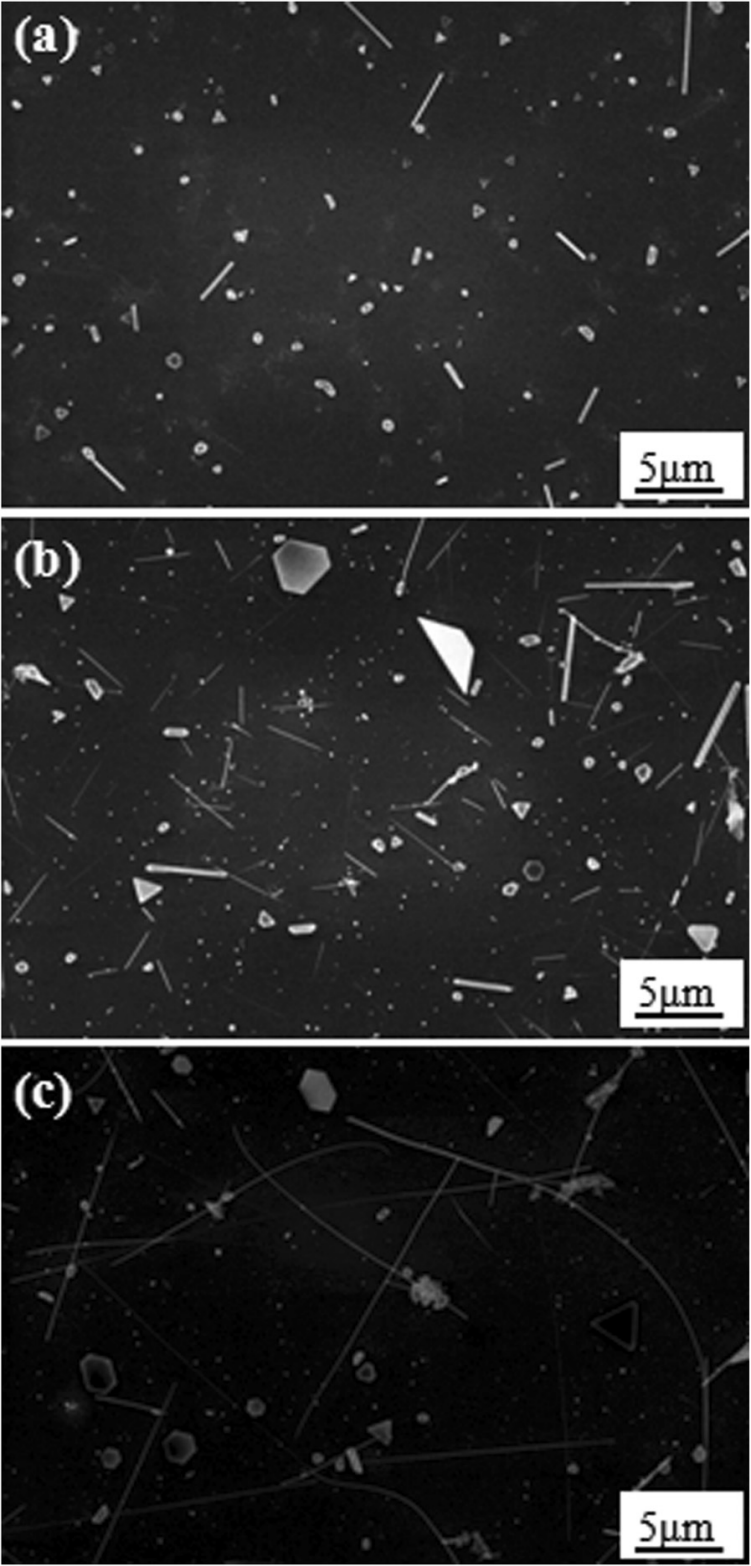
**SEM images of CoSi nanowires.** At vapor pressures of **(a)** 100, **(b)** 300, and **(c)** 500 Torr, respectively.

The second processing parameter we investigated was the vapor pressure. Figure 3a,b,c show our SEM studies for 100, 300, and 500 Torr, respectively. It turns out that CoSi nanowires grew particularly well at the reaction pressure of 500 Torr. In this experiment, the higher the vapor pressure, the longer the nanowires grown. Additionally, with the increasing vapor pressure, the number of nanoparticles reduces, but the size of the nanoparticles increases.

**Figure 4:**
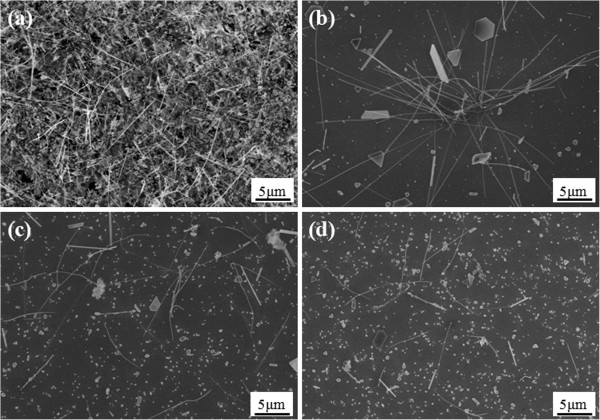
**SEM images of CoSi nanowires.** At gas flow rates of **(a)** 200, **(b)** 250, **(c)** 300, and **(d)** 350 sccm, respectively.

For the synthesis of cobalt silicide nanowires, the third and final processing parameter we studied was the gas flow rate. We conducted experiments at the gas flow rate of 200, 250, 300, and 350 sccm, obtaining the corresponding results shown in Figure 4a,b,c,d, respectively. It can be found in the SEM images of Figure 4 that at 850°C ~ 880°C, the number of CoSi nanowires reduced with the increasing gas flow rate; thus, more CoSi nanowires appeared as the gas flow rate was lower.

**Figure 5:**
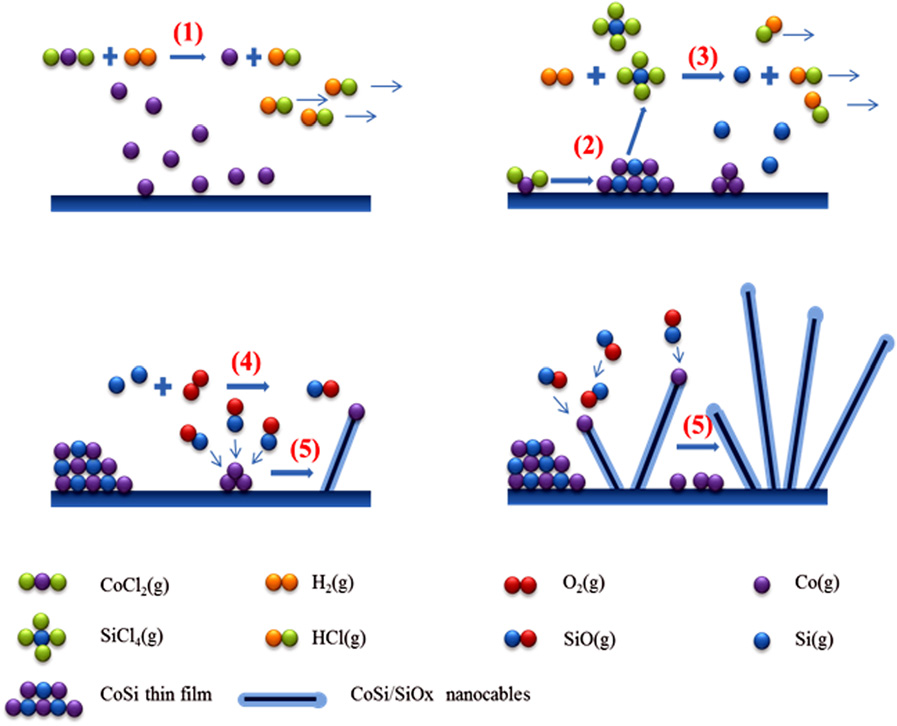
**The schematic illustration of the growth mechanism.** (1) CoCl_2_(g) + H_2_(g) → Co(s) + 2HCl(g), (2) 2CoCl_2_(g) + 3Si(s) → 2CoSi(s) + SiCl_4_(g), (3) SiCl_4_(g) + 2H_2_(g) → Si(g) + 4HCl(g), (4) 2Si(g) + O_2_(g) → 2SiO(g), and (5) Co(solid or vapor) + 2SiO(g) → CoSi(s) + SiO_2_(s).

The growth mechanism of the cobalt silicide nanowires in this work is of interest. Figure 5 is the schematic illustration of the growth mechanism, showing the proposed growth steps of CoSi nanowires with a SiOx outer layer. When the system temperature did not reach the reaction temperature, CoCl_2_ reacted with H_2_ (g) to form Co following step (1) of Figure 5:

$${\mathrm{CoCl}}_{2}\left(g\right)+H_{2}\left(g\right)\to \mathrm{Co}\left(s\right)+2\mathrm{HCl}\left(g\right)$$

The Co atoms agglomerated to form Co nanoparticles on the silicon substrate. When the system temperature reached the reaction temperatures, 850°C ~ 880°C, CoCl_2_ reacted with the silicon substrate to form a CoSi thin film and SiCl_4_ based on step (2) of Figure 5:

$$\begin{array}{ll} 2{\mathrm{CoCl}}_{2}\left(g\right) & +3\mathrm{Si}\left(s\right)\to 2\mathrm{CoSi}\left(s\right)\\ & \;+{\mathrm{SiCl}}_{4}\left(g\right),T=850^{\circ }C\sim 880^{\circ }C \end{array}$$

The SiCl_4_ product then reacted with H_2_(g) to form Si(g) following step (3) of Figure 5:

$${\mathrm{SiCl}}_{4}\left(g\right)+2H_{2}\left(g\right)\to \mathrm{Si}\left(g\right)+4\mathrm{HCl}\left(g\right)$$

The Si here reacted with either residual oxygen or the exposed SiO_2_ surface to form SiO vapor from step (4) of Figure 5[30]:

$$2\mathrm{Si}\left(g\right)+O_{2}\left(g\right)\to 2\mathrm{SiO}\left(g\right)\mathrm{or}\mathrm{Si}\left(g\right)+{\mathrm{SiO}}_{2}\left(g\right)\to 2\mathrm{SiO}\left(g\right)$$

The SiO vapor reacted with Co nanoparticles via vapor-liquid–solid mechanism. Consequently, CoSi nanowires with a SiOx outer layer were grown through step (5) of Figure 5[30]:

$$\mathrm{Co}\left(\mathrm{solid}\;\mathrm{or}\;\mathrm{vapor}\right)+2\mathrm{SiO}\left(g\right)\to \mathrm{CoSi}\left(s\right)+{\mathrm{SiO}}_{2}\left(s\right)$$

When the substrate temperature was at 880°C ~ 900°C, CoCl_2_ reacted with the silicon substrate to form Co_2_Si nanoparticles and SiCl_4_:

$$\begin{array}{ll} 2{\mathrm{CoCl}}_{2}\left(g\right) & +3\mathrm{Si}\left(s\right)\to 2{\mathrm{Co}}_{2}\mathrm{Si}\left(s\right)\\ & \;+{\mathrm{SiCl}}_{4}\left(g\right),T=880^{\circ }C\sim 900^{\circ }C \end{array}$$

The SiCl_4_ also reacted with CoCl_2_ to form Co_2_Si, transforming Co_2_Si nanoparticles to Co_2_Si nanowires through self-catalysis:

$$\begin{array}{ll} 2\mathrm{CoC}l_{2}\left(g\right) & +\mathrm{SiC}l_{4}\left(s\right)\to 2Co_{2}\mathrm{Si}\left(s\right)\\ & \;+4Cl_{2}\left(g\right),T=880^{\circ }C\sim 900^{\circ }C \end{array}$$

**Figure 6:**
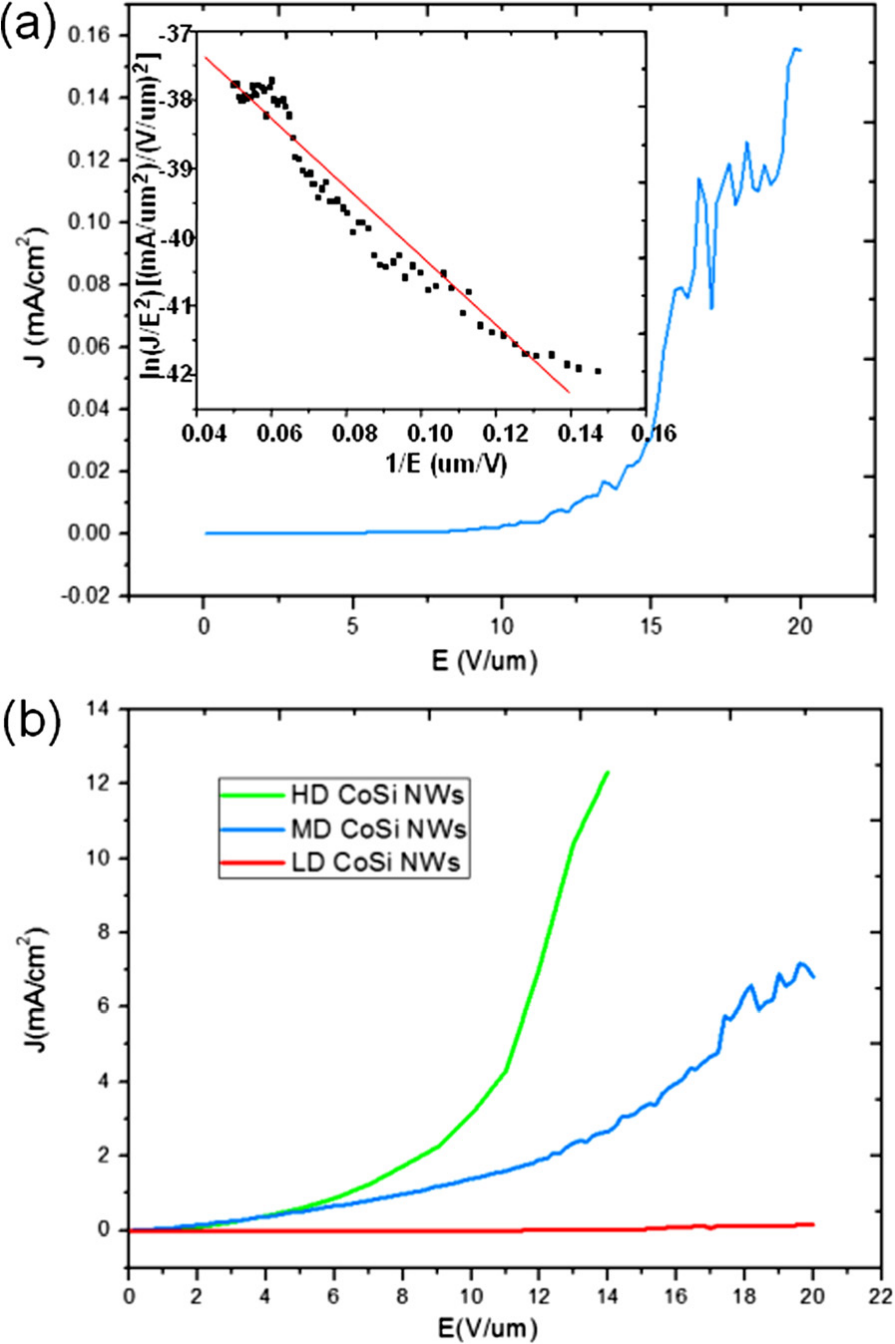
**Field emission analysis. (a)** The field emission plot of CoSi NWs. The inset in (a) shows the corresponding ln(*J*/*E*^2^) − 1/*E* plot. **(b)** The field emission plot of CoSi NWs with different densities.

In addition to understanding the growth behaviors of the cobalt silicide nanowires, we explored their physical properties and etched away the oxide shell before measurements. Figure 6 shows the field emission measurements for CoSi NWs. Figure 6a is the plot of the current density (*J*) as a function of the applied field (*E*) with the inset of the ln(*J*/*E*^2^) − 1/*E* plot. The sample was measured in a vacuum chamber pump to approximately 10^−6^ Torr. According to the Fowler-Nordheim plot and the Fowler-Nordheim equation:

$$J=\left(A{ß}^{2}E^{2}/\varphi \right)\exp\left(-B\varphi^{3/2}/\mathrm{ßE}\right),$$

where *J* is the current density, *E* is the applied electric field, and *φ* is the work function; for CoSi, *φ* is 4.7 eV. *A* and *B* are constants, corresponding to 1.56 × 10^−10^ (*A* (eV)/*V*^−2^) and 6.83 × 10^9^ (*V* (eV)^−3/2^ m^−1^), respectively. The field enhancement *ß* has been calculated to be 1,384 from the slope of ln(*J*/*E*^2^) = ln(*Aß*^2^/*φ*) − *Bφ*^3/2^/*ßE*, proving that CoSi NWs are promising emitters. Also, the higher the density of CoSi NWs, the better the field emission property as shown in Figure 6b. The outstanding field emission properties of CoSi NWs are attributed to their metallic property and special one-dimensional geometry.

## Conclusions

In this study, using a CVD method, we have synthesized cobalt silicide nanowires of two different phases, which are CoSi NWs and Co_2_Si NWs, respectively. Effects of some processing parameters, including the temperature, gas flow rate, and pressure, were investigated; for example, the number of CoSi nanowires shows a decreasing trend with the increasing gas flow rate. Also, the growth mechanism has been proposed. Electrical measurements demonstrate that the CoSi nanowires are potential field-emitting materials.

## Competing interests

The authors declare that they have no competing interests.

## Authors’ contributions

CML and KCL conceived the study and designed the research. CML conducted the experiments. CML, HFH, and KCL wrote the manuscript. All authors read and approved the final manuscript.
